# Brazos Valley Medical Association

**Published:** 1897-05

**Authors:** 


### Third Semi-Annual Meeting of the Brazos Valley Medical Association, Cameron, Texas, May 11th and 12th, 1897.

"Like as a plank of driftwood,
Tossed on the watery main,
One plank encounters,
Meets, touches, parts again."

"Thus 'tis with friends forever,
On life's uncertain sea,
They meet, and greet, and sever,
Drifting eternally."

PROGRAMME.

1. Paper—Infant Feeding; Its Results—Dr. G. M. Abney, Franklin. Discussion—Dr. W. W. Greer, Cameron; Dr. Geo. R. Tabor, Bryan.

2. Paper—Malaria Hæmaturia—Dr. W. B. Briggs, Easterly. Discussion—Dr. D. Monroe, Cameron; Dr. Daniel Parker, Calvert.

3. Paper—Third Stage of Labor; Its Sequellæ—Dr. B. F. Watkins, Bryan. Discussion—Dr. W. F. Sharp, Davilla; Dr. F. R. Collard, Wheelock.

4. Paper—Inflammation—Dr. D. L. Peeples, Navasota. Discussion—Dr. Thos. A. Pope, Cameron; Dr. T. G. Curry, Bremond.

5. Paper—To be supplied—Dr. D. Monroe, Cameron. Discussion—Dr. D. H. Bailey, Branchville; Dr. G. H. Richardson, Hayes.

6. Paper—LaGrippe—Dr. W. W. McDonald, Easterly. Discussion—Dr. R. W. Wallis, Rockdale; Dr. O. T. Lewis, Welborn; Dr. C. T. Doremus, Hearne.

7. Paper—Diseases Incident to Pregnancy—Dr. J. W. Hudson, Milano. Discussion—Dr. J. M. Nicks, Stone City; Dr. W. H. Harrison, Bryan. Dr. W. A. Smith, Hearne.

8. Paper—Eczema—Dr. R. W. Nobles, Temple. Discussion—E. Brittain, Bremond; Dr. H. Fountain, Bryan.

9. Paper—Membranous Laryngitis—Dr. J. P. Oliver, Caldwell. Discussion—Dr. I. P. Sessions, Rockdale; Dr. J. H. Brewton, Franklin; Dr. A. G. Barnhill, Sebasta.

10. Paper—To be supplied—Dr. W. W. Greer, Cameron. Discussion—Dr. R. S. Carroll, Calvert; Dr. John D. Porter, Gause; Dr. L. L. Todd, Harvey.

11. Paper—Dysentery—Dr. A. J. Ellzey, Lilac. Discussion—Dr. A. Kobra, Rockdale; Dr. W. J. Adderhold, Millican; Dr. W. P. Gilstrap, Wheelock.

12. Paper—To be supplied—Dr. E. N. Shaw, Cameron. Discussion—Dr. W. C. Taylor, Branchville; Dr. L. M. Bassett, Hearne; Dr. J. M. Sales, College Station.

Voluntary papers and report of cases.

H. W. Cummings, President.

E. Brittain, Secretary.

---

Hearne, Texas, 1897.

Dear Doctor:—I have the pleasure of extending to you an invitation to the Third Semi-Annual Meeting of the Brazos Valley Medical Association, which convenes at Cameron, Texas, May 11 and 12, 1897.

It is needless to suggest to you the great advantage of active society work; nor to remind you of the great need of such work in the profession of our district, as well as our State. But in order to accomplish the ends desired, it becomes imperative to urge upon you the necessity of your individual and personal aid, reminding you that no organization can live and thrive when its members are indifferent to its welfare.

The coalition of mind with mind and of thought with thought, stimulates and energizes the mental digestive powers, making one a truer and better physician, and is the only means to accomplish for ourselves any of the great results which are being done for the relief and benefit of human ills.

Trusting, doctor, that you will realize your obligation to the profession, and will lend your influence and aid in making this Association one which will do much toward the upbuilding of scientific medicine in our district, I am

Sincerely yours,
H. W. Cummings, President.

